# Linguistic and Clinical Validation of the Polish Version of the Walking Impairment Questionnaire

**DOI:** 10.3390/medsci14040437

**Published:** 2026-07-26

**Authors:** Mikołaj Maga, Paweł Kaczmarczyk, Agnieszka Śliwka, Paulina Kłapacz-Krężel, Jakub Krężel, Mateusz Blukacz, Paweł Maga

**Affiliations:** 1 Clinical Department of Angiology and Internal Medicine, University Hospital in Krakow, Ul. Jakubowskiego 2, 30-688 Kraków, Poland; pawel.kaczmarczyk@uj.edu.pl (P.K.); paulina24k@gmail.com (P.K.-K.); jakub.krezel@gmail.com (J.K.); pawel.maga@uj.edu.pl (P.M.); 2 Department of Rheumatology and Immunology, Medical Faculty, Jagiellonian University Medical College, 30-688 Krakow, Poland; 3 Department of Angiology, Medical Faculty, Jagiellonian University Medical College, 30-688 Krakow, Poland; 4 Department of Rehabilitation in Internal Medicine, Faculty of Health Sciences, Jagiellonian University Medical College, 31-066 Krakow, Poland; agnieszka.sliwka@uj.edu.pl; 5 Department of Environmental Health, Faculty of Health Sciences, Jagiellonian University Medical College, 30-688 Krakow, Poland; mateusz.blukacz@uj.edu.pl

**Keywords:** peripheral arterial disease, quality of life, walking impairment questionnaire, validation

## Abstract

Background: Intermittent claudication is one of the most common symptoms of peripheral arterial disease. The Walking Impairment Questionnaire (WIQ) is frequently used to assess patients suffering from intermittent claudication as it measures walking distance, speed, and ability to climb stairs. Although the questionnaire has been translated and employed in different countries, no validated Polish version is available yet. The aim of this study was to translate and validate the Polish version of the WIQ. Methods: One hundred patients with IC who qualified for endovascular treatment were enrolled. WIQ translation and clinical validation was performed. The correlations among the WIQ, the two quality of life questionnaires (VascuQol-25 and SF-36), and objective walking distances in a 6-minute walking test (6 MWT) were calculated to determine the validity. Results: Correlations were observed among all WIQ domains, as well as between the total WIQ score and both initial claudication distance (InCD) and maximum walking distance (MWD), across all domains and 6 MWT parameters. The WIQ correlated with all domains of both VascuQol and SF-36. Test–retest reliability, as measured using the ICC, was 0.90. The internal consistency determined using Cronbach’s alpha was 0.94 for the total WIQ score. Conclusions: This study demonstrates that the Polish version of the WIQ, using the European metric system, is a reliable and clinically relevant tool for assessing walking impairment in patients with intermittent claudication.

## 1. Introduction

Peripheral arterial disease (PAD) is one of the most common civilization-related global burdens, with a prevalence reaching up to 236 million patients, and it is estimated to be present in 10% up to 26% of the adult population [1,2]. In Poland, 4000–5000 hospitalizations are performed annually due to PAD, and over 10,000 major amputations are carried out annually due to CLTI in the course of PAD [3]. One of the most common early manifestations, intermittent claudication (IC), is described as cramping, aching, or pain in the calves, thighs, or buttocks. This reproducible discomfort or fatigue in the muscles of the lower extremity occurs with exertion and is relieved within 10 min of rest [4]. It often leads to reduced mobility, limited physical functioning, and a reduced quality of life (QoL) [5]. Usually, treadmill tests are performed to assess the severity of walking impairment, where the distance of walking is measured until the onset of pain or pain intolerance development [6]. However, treadmill testing is time-consuming, often not available, and inconvenient. Furthermore, it does not represent the walking ability of patients during daily life [7]. For this reason, Regensteiner et al. developed the Walking Impairment Questionnaire (WIQ) [8]. The WIQ is a short, easy-to-fill questionnaire that measures walking distance, speed, and ability to climb stairs. These three domains represent common daily activities that are frequently impaired in patients with symptomatic PAD and contribute to the degree of walking impairment. It has been widely adopted in both clinical practice and research and validated in multiple condition sets, making it one of the most commonly used disease-specific PROMs for assessing walking limitation [9]. Its results have been shown to be closely related to quantitative measures of arterial disease such as initial and absolute claudication distances (ICD and ACD, respectively) and maximum walking time (MWT) of the patient with intermittent claudication [10] and have also been used to assess the efficacy of clinical interventions [11]. The tool can be administered by interview or completed by the patient, with no significant impact on responses [12,13]. The questionnaire has been translated into multiple languages and employed in different countries, but only a few linguistic versions underwent the validation process (Dutch, Spanish, American Hispanic, Japanese, Swedish, Chinese, Brazilian, and Lithuanian) [7,14,15,16,17,18,19,20]. The Polish version remains unverified.

We aim to translate the WIQ into the Polish language and validate the Polish version, determining the reproducibility of the WIQ scores and its correlation with other objective indices such as the ankle–brachial index (ABI), toe–brachial index (TBI), and the six-minute walk test (6 MWT) to combine declared symptoms with the objective unbiased values. 

## 2. Materials and Methods

Study design

In this prospective validation study, the data obtained in the three WIQ domains (distance, velocity, and stairs) were compared with the data obtained with the Medical Outcome Study Short-Form 36 (SF-36) questionnaire and the VascuQol-25 questionnaire to analyze the validity of the construct. Both questionnaires were previously validated for the Polish population [21,22]. The objective indices of the patients, including the ABI, TBI, initial claudication distance (InCD), and maximum walking distance (MWD), were measured at the beginning of the study.

Patients

In total, 100 consecutive patients with symptomatic PAD admitted for endovascular intervention to the Clinical Department of Angiology and University Hospital in Krakow (Poland) who met the inclusion criteria (stage 3 of the Rutherford classification, age 45–70 years, ability to perform walking test) were enrolled. All of them were Polish. Exclusion criteria were selected to ensure that participants could walk safely and that the PAD-induced claudication was the limiting factor for walking performance without the impact of other comorbidities on the obtained results (chronic limb-threatening ischemia, stage III and IV CCS, chronic obstructive pulmonary disease, comorbidities limiting walking ability not related to lower limbs ischemia, and cognitive functions disorders limiting capability to follow the instructions and fill out the questionnaires).

Power and sample size calculations

The sample size was calculated using the alpha level of 0.05, a statistical power of 80%, and a confidence level of 95% based on the previously published data. Using these assumptions, 88 patients would be needed; however, accounting for a 10% loss to follow-up or missing data, the final sample size was estimated to be 100 participants [20].

Polish version of WIQ

The WIQ is a patient-reported outcome measure, disease-specific questionnaire. It contains 14 items that are divided into three subscales (distance, speed, and stair) and an overall score. Participants answer each item on a scale from 0 for “unable to do” to 4 for “no difficulty”. The translation was performed according to the Beaton’s guidelines describing the process of cross-cultural adaptation of self-report measures [23]. First, the original English questionnaire was translated into the Polish version by three bilingual Polish translators including two specialist in angiology and one psychologist who were blinded to other the translators’ versions. After their work, they discussed the best final version. The acquired Polish version was translated back to the English version by another three bilingual translators who were medical students and their native language was English. Any disagreements between translators were resolved by voting, and the options with the major support were chosen. In the next step, cognitive debriefing and pilot testing were performed with 15 patients. As there were no issues reported by patients, the version of the translated WIQ was accepted as the final. After discussion among the authors and the introduction of several minor changes, the final Polish version was selected. Thereafter, the participants filled out the translated WIQ questionnaire under the supervision of a single previously trained examiner. The summarized value and scores for each domain of the WIQ were calculated [12].

The SF-36 questionnaire

The 36-Item Medical Outcomes Study Short Form (SF-36) is a self-administered questionnaire that contains 36 items. It measures health on eight multi-item dimensions, covering physical function, social functioning, role limitations due to physical problems, role limitations due to emotional problems, mental health, vitality, pain, and general health perception [24,25]. It is the most commonly used generic health-related quality of life (HRQoL) assessment tool worldwide. Due to the extensive use and experience across patients and the general population, the generic SF-36 questionnaire also allows the comparison of perceived health status across various disorders and with the general population, including intermittent claudication [26]. The Polish version of the questionnaire has been validated and is often used in life quality studies of different groups of patients [21,27]. The SF-36 data were filled out by participants under the supervision of a single previously trained examiner. Scores were calculated for each of the subscales. The total score on each SF-36 subscale ranges from 0 to 100. A higher score indicates a better HRQoL.

The VascuQoL-25

The VQ-25 is a disease-specific questionnaire designed to assess HRQoL in symptomatic PAD regardless of disease severity (both in IC and in chronic limb-threatening ischemia) [28]. The questionnaire comprises six items that cover different HRQoL aspects (two activity items and one item for each of PAD symptoms, pain, emotional, and social functioning). Each item refers to lower limb circulatory problems using a 2-week recall period and has a four-point response scale. Item responses are summarized to generate an overall score ranging from 6 (worst HRQoL) to 24 (best HRQoL) [29]. In this study, the Polish validated version of the questionnaire was used [22].

Six-minute walk test (6 MWT)

The 6 MWT measures walking endurance with excellent test–retest reliability in patients with PAD [30,31]. Following a standardized protocol, participants walked up and down a hallway for 6 min after instructions to cover as much distance as possible. The distance completed during in 6 min was recorded. The initial claudication distance measured the distance in meters walked before the onset of claudication. Maximum walking distance was determined by the total distance walked due to claudication. The participants were instructed to notify the staff when they first experienced claudication and when they had to stop walking due to claudication.

Other variables

During the admission procedure, a detailed vascular-focused medical interview was conducted, including comorbidities, medications taken, and smoking status. Additionally, ABI and TBI measurements were performed with the use of a Doppler Bidop ES-100 V3 equipped with a PG-21 photoplethysmography probe [32].

Reliability of the WIQ

Reliability refers to the repeatability, stability, or internal consistency of a questionnaire. One of the most common ways to demonstrate this is using the Cronbach’s α statistic. This statistic uses inter-item correlations to determine whether constituent items measure the same domain [33]. Internal consistency measures the degree of homogeneity of a scale based on the correlation between each of the items and the correlation between the items and the total score. It is usually used to report Cronbach’s α statistic for the separate domains within a questionnaire rather than for the entire questionnaire.

Internal consistency and test–retest reliability were calculated to determine the WIQ’s reliability. The reliability was estimated by performing the questionnaire twice by the same participants. The absolute agreement intraclass correlation coefficient based on a mixed model with a 95% confidence interval was used in the test-retest reliability assessment. For the test-retest reliability assessment, 20 randomly selected participants were enrolled, who filled out WIQ two times, separated by 2–5 days. This interval was considered sufficiently long to minimize the likelihood that patients would recall their previous responses, while remaining short enough to avoid a significant treatment effect, as there were no any new interventions performed in the meantime.

Validity of the WIQ

There are several different types of validity [34]. Content validity refers to the expert opinion on whether the scale items represent the proposed domains or concepts that the questionnaire is intended to measure. Convergent (or concurrent) and discriminant validity must also be demonstrated by correlating the measure with related and/or dissimilar established measures with proven validity. In this study, the correlation between WIQ and the initial claudication distance and the maximum walking distance assessed using a six-minute walk test was calculated.

Construct validity relates to the degree to which the items in the questionnaire represent the underlying conceptual structure. If a golden standard is absent, the construct validity is determined, and the WIQ is compared with other tests that measure the same construct. In this study, we compared the WIQ with two general quality of life questionnaires, the SF-36 and the VQ-6, measuring the same construct: walking impairment. The correlations were determined using Spearman correlation coefficients.

Ethical aspects

The work described in our article has been carried out in accordance with The Code of Ethics of the World Medical Association (Declaration of Helsinki) for experiments involving humans, and the proper consent for this study has been obtained from the Bioethics Board of the Regional Medical Chamber in Krakow (66/KBL/OIL/2017). All of the study participants provided informed consent. 

## 3. Results

### 3.1. Study Population

In total, 100 patients with a mean age of 66.39 years who had been admitted to the Angiology Department with short-distance IC in the course of PAD to undergo endovascular treatment were enrolled in this study. There was a slight bias for male patients (61.54%). Most of them had a history of smoking and hypertension. Their detailed characteristics are described in Table 1.

All participants underwent the 6 MWT, but 25 of them had to stop and were unable to continue due to severe claudication. The mean MWD was 251.49 m, while the InCD was 90.44 m. Only one patient needed additional equipment during 6 MWT (crutch). Although 42% of the participants reported dyspnea after the walking test, this was rather a subjective feeling as the O_2_ saturation remained comparable to pretest levels (Table 2).

### 3.2. Reliability and Internal Consistency

**Figure 1:**
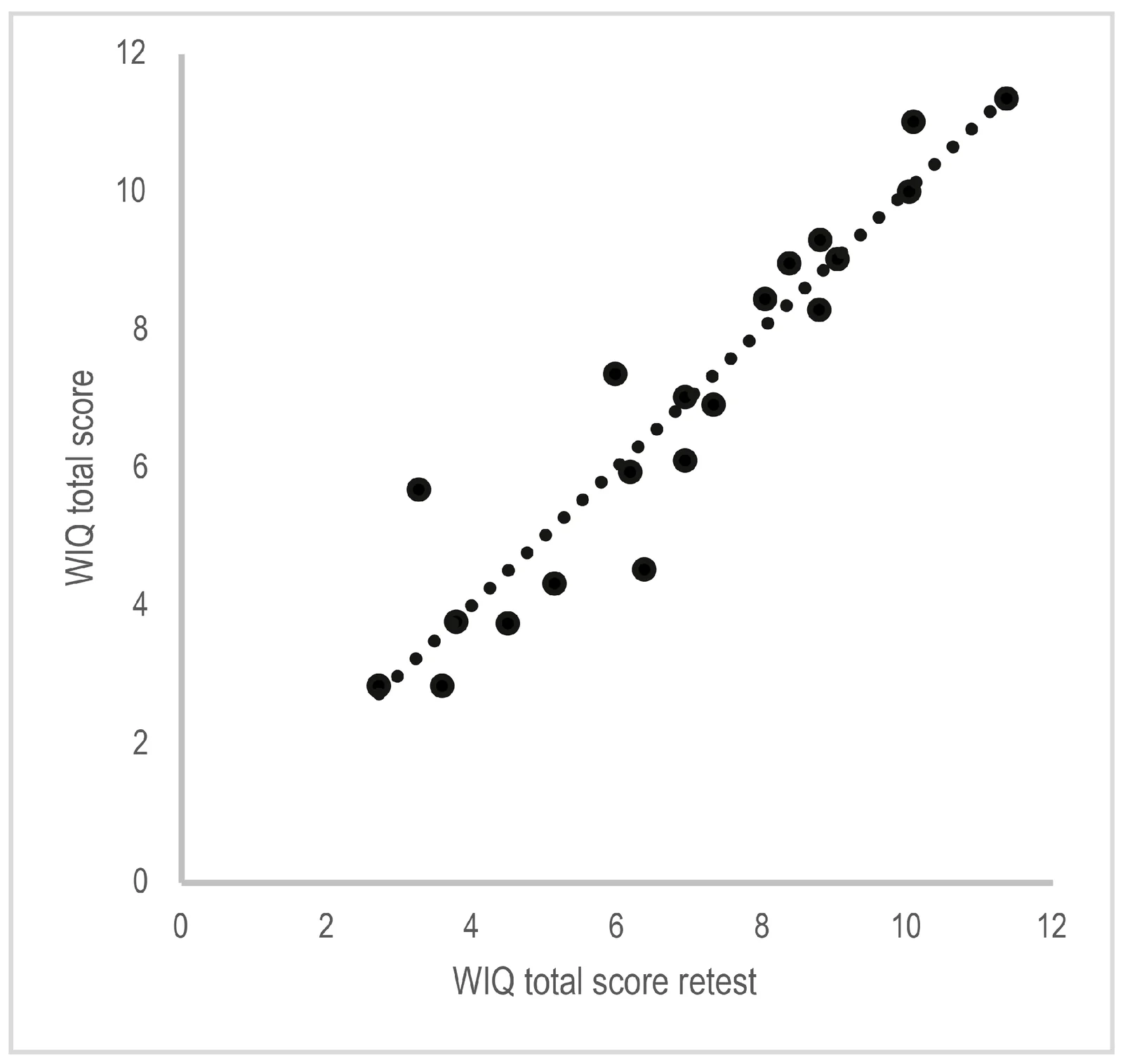
WIQ total score test–retest results (n = 20).

All participants completed the WIQ, giving a mean total score of 6.17 (SD 3.03). The internal consistency scores were 0.94 for the total score, 0.89 for the distance, 0.83 for the speed, and 0.90 for the stair climb, indicating sufficient homogeneity in all domains and the entire questionnaire itself. In the test–retest analysis performed by 20 randomly picked participants, the ICC for the individual WIQ domains were 0.83 (speed), 0.84 (stair climbing), and 0.87 (distance), while the ICC for the total WIQ score was the highest at 0.90, implicating strong reliability (Table 3 and Figure 1).

### 3.3. Concurrent Validity

**Figure 2:**
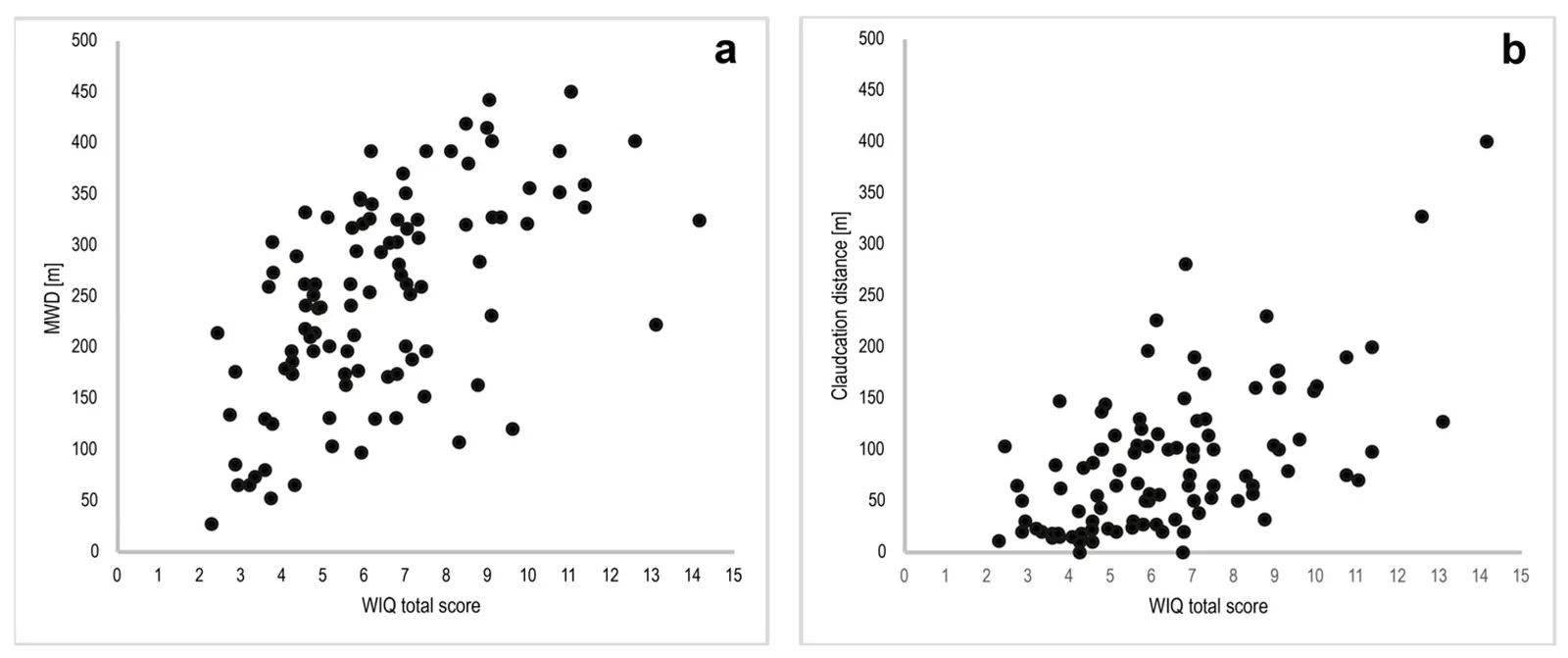
The total WIQ score plotted against the MWT (**a**) and InCD (**b**).

There is a significant correlation among all WIQ domains, as well as its total score, and both InCD and MWD with *p* < 0.001 for all domains, and the 6 MWT parameters with the exception of stair climbing and InCD with *p* = 0.001 (Figure 2).

The clinical blood flow parameters represented by ABI and TBI correlated not only with the speed domain (respectively *p* = 0.005 and 0.004) but also with the total score (*p* = 0.037 and 0.04) although the strength of this dependence was smaller (Table 4).

### 3.4. Construct Validity

The construct validity was based on comparing WIQ results to VascuQol-25 and SF-36 results, which were also divided into domains. As presented in Table 5, WIQ correlated with all domains of both VascuQol (rho 0.45–0.68) and SF-36 (rho 0.31–0.67). The WIQ total score also correlated with the SF-36 (rho 0.64 *p* < 0.001) total score. The highest Spearman correlation coefficient was observed in the WIQ total score and VascuQol-25 activities domain (rho 0.68, *p* < 0.001), while the weakest was noted for WIQ distance and SF-36 general health perception (rho 0.31, *p* = 0.002).

### 3.5. WIQ Distance vs. 6 MWT Results

The WIQ total scores divided into terciles also showed a relationship to 6 MWT parameters, as the higher WIQ scores corresponded to a longer maximal walking distance as well as initial claudication distance (Table 6).

In the WIQ distance domain, 58% of the participants declared a shorter maximal walking distance than achieved in the 6 MWT. In 14 (24.1%) of them, the difference did not exceed 25%. In 25 (43.1%), the difference was between 25 and 50%. In 19 (32.8%) participants, the difference was greater than 50%. Among participants who declared a longer distance than finally achieved in 6 MWT, a majority (n = 24; 57.2%) walked over 50% longer than in the WIQ. Overestimation up to 25% was present in nine participants (21.4%), and nine (21.4%) participants had overestimation in the range of 25–50%.

In 64 participants, the real claudication distance in 6 MWT was shorter than what was declared in the WIQ. Of these, in 46.9%, the difference exceeded 50 m. In 67.2% cases, the difference was greater than 25%. In the remaining 36 participants, the WIQ claudication distance was longer than the initial claudication distance. In 15 cases (41.7%), it was greater than 50 m. In 21 cases (58.3%), it was over a 25% difference.

## 4. Discussion

Even though the WIQ was already translated into 56 languages, not all versions have been validated [35]. To compare responses across populations of different languages and/or cultures, researchers must ensure that the questionnaires in different languages assess the equivalent construct with an equivalent metric [36]. That is why the reliability of a questionnaire, considered as the consistency of the survey results, needs to be provided for each language version. After the translated questionnaire items pass the preliminary pilot testing and subsequent revisions, they should be a subject of the validation [37].

The Polish version of the WIQ is available to download from the questionnaire distributor, but its linguistic, cultural, or clinical validations were never performed. Despite the lack of validation, this tool was used in a Polish facility in a recent SUPERSURG trial [38]. In addition, E. Rosloniec et al. even used it to validate the Polish version of another questionnaire [39]. As there are no available data on the methodology used for the existing version, the translation process for this validation has been performed from the very beginning to ensure proper validation.

Although the WIQ is an effective condition-specific instrument for assessing mobility in individuals with intermittent claudication (IC), it does not capture broader aspects of quality of life (QOL) associated with peripheral arterial disease (PAD). Therefore, the choice of using either a single patient-reported outcome measure (PROM) or a combination of PROMs should be determined by clinicians or researchers based on the specific objectives of their assessment [9]. In accordance with Mahe G. et al. [40], the self-completion of the WIQ increases significantly the risk of errors biasing the obtained results, but neither this study nor other studies confirmed these findings.

Previous studies have reported moderate to strong correlations between WIQ scores on the American version of the WIQ and walking performance as measured using treadmill tests in patients with intermittent claudication [10]. Similar dependences were observed by Verspaget et al. using a Dutch version of the questionnaire [20]. Those results are in agreement with our observations, as WIQ declared distances correlated with the 6 MWT results. However, our study showed that the precise estimation of the maximum walking distance may be difficult for patients, confirming the previous observation reported by Grzela et al. [41]. Even though overestimation was more common (57%), significant differences (over 50%) were way more often declared as underestimations of the claudication distance. This is the first time such a detailed analysis has been performed between WIQ-declared walking distances and real-life walking data. As the use of 6 MWT instead of treadmill testing was proven to be more accurate due to the similar way and environment of daily activity, we decided to use this method of claudication distance assessment [42]. 

The obtained results regarding test–retest reliability present a slightly higher ICC compared to the Dutch, Brazilian, and Chinese versions [15,17,20] and an ICC comparable to the Lithuanian version [16], despite a lower sample size of this subanalysis. This proves that the Polish translation is clear for the patients and was not vulnerable to the misunderstanding bias. For the construct validity, two different types of QoL questionnaires were chosen. SF-36 is one of the most commonly used generic questionnaires, and the obtained results were in accordance with the Dutch validation and superior compared to the Brazilian validation. The second picked Qol questionnaire was VascuQol, which is a disease-specific questionnaire used for PAD patients. This is the only study that incorporated this questionnaire for WIQ validation, and the results presented were even slightly more solid than those for SF-36, which is probably due to the characteristics of the questionnaires. 

Our translation, similar to other European versions, took into account the differences between metric and US imperial units. The original WIQ included block-based distance estimations, while in Poland, there is no standard definition or distance related to a single block. We assumed that one block would be equal to the distance of approximately 100 m, as in imperial units it mostly refers to 300 feet. 

The study has limitations; however, it was designed to minimize their impact on the results obtained. The number of participants is relatively low but was established based on power and sample size calculations. Participants were only classified as Rutherford 3 with a relatively short distance of claudication requiring endovascular intervention, while Rutherford 1 and 2 patients were not evaluated. Last but not least, the test–retest analysis was performed on only 20% of the participants, but they were randomly chosen from the whole study population. The sample size of this subgroup was established based on previous studies, but not proper power and sample size calculations. The interval was not fully consistent, and the difference in the time gap as well the circumstances (home vs. hospital setting) differed, potentially affecting responses. We believe that the mentioned limitations did not bias the study in a significant way. 

## 5. Conclusions

To conclude, the Polish version of the Walking Impairment Questionnaire is highly repeatable in the Polish population. Its results correlate with general health and disease-specific life quality questionnaires, such as SF-36 and Vascu-Qol-25. They also reflect differences in objective clinical parameters such as intermittent claudication distances and peripheral blood pressure, representing blood supply.

## Figures and Tables

**Table 1:** Detailed characteristics of the patients enrolled in the study.

General Information	
Age (years)—mean (SD)	66.39 (±9)
Male—n (%)	64 (61.54%)
Body mass index—mean (SD)	25.92 (±5.25)
Hypertension—n (%)	76 (73.08%)
Diabetes mellitus—n (%)	38 (36.54%)
Coronary artery disease—n (%)	40 (38.46%)
Chronic heart failure—n (%)	8 (7.69%)
History of myocardial infarction—n (%)	19 (18.27%)
History of percutaneous coronary intervention—n (%)	26 (25%)
History of stroke or transient ischemic attack (TIA)—n (%)	11 (10.58%)
Atrial fibrillation—n (%)	7 (6.73%)
Chronic kidney disease—n (%)	5 (4.8%)
Chronic obstructive pulmonary disease—n (%)	8 (7.69%)
Asthma—n (%)	5 (4.8%)
Cigarette smoking	
Former smokers—n (%)	66 (63.46%)
Current smokers—n (%)	28 (26.92%)
Never smoked—n (%)	8 (7.69%)
Clinical vascular parameters	
Treated leg preoperative ABI—mean (SD)	0.47 (±0.21)
Treated leg preoperative TBI—mean (SD)	0.27 (±0.2)
Total score WIQ—mean (SD)	6.17 (±3.18)

**Table 2:** Results of the 6-minute walking test (6 MWT).

6 MWT Results	
Maximum walking distance [m] (SD)	251.49 (±101.27)
Initial claudication distance [m] (SD)	90.44 (±76.13)
Patients not finishing due to severe claudication (n)	25 (25%)
Presence of dyspnea after 6 MWT (n)	42 (42%)
Dyspnea level on the Borg scale after 6 MWT (SD)	2.52 (±1.19)
Presence of fatigue after 6 MWT (n)	78 (78%)
Fatigue level on the Borg scale after 6 MWT (SD)	2.82 (±1.85)
Heart rate before 6 MWT (SD)	75.90 (±12.38)
Heart rate after 6 MWT (SD)	85.08 (±14.92)
O_2_ saturation before 6 MWT (SD)	96.4 (±1.52)
O_2_ saturation after 6 MWT (SD)	96.62 (±1.75)

**Table 3:** Test–retest reliability values (n = 20).

WIQ	Baseline Mean (SD)	Retest Mean (SD)	Change of Scores Mean (SD)	Test–Retest (ICC)	95% CI
Distance	10.06 (4.58)	9.23 (4.63)	0.82 (2.30)	0.87	0.68–0.95
Speed	4.29 (3.00)	4.66 (2.80)	−0.35 (1.69)	0.83	0.60–0.94
Stair climbing	4.18 (2.83)	4.18 (2.32)	0.00 (1.50)	0.84	0.61–0.94
Total score	6.17 (3.03)	6.02 (2.87)	0.16 (1.33)	0.90	0.75–0.96

**Table 4:** Spearman correlations of concurrent validity between WIQ results and initial claudication distance (InCD), maximum walking distance (MWD), ankle–brachial index (ABI), and toe–brachial index (TBI).

WIQ	MWD	*p*	InCD	*p*	ABI	*p*	TBI	*p*
Distance	0.53	<0.0001	0.54	<0.0001	0.18	0.0844	0.18	0.0816
Speed	0.45	<0.0001	0.43	<0.0001	0.29	0.0045	0.29	0.0040
Stair climbing	0.47	<0.0001	0.39	0.0001	0.14	0.1894	0.08	0.4215
Total score	0.54	<0.0001	0.52	<0.0001	0.21	0.0374	0.21	0.0420

**Table 5:** Construct validity. Spearman correlation coefficients among WIQ, VascuQol-25, and SF-36 (n = 100). ^a^ *p* < 0.0001, ^b^ *p* < 0.001.

	Distance	Speed	Stair Climbing	Total Score
VascuQol-25				
Activities	0.60 ^a^	0.54 ^a^	0.65 ^a^	0.68 ^a^
Symptoms	0.53 ^a^	0.49 ^a^	0.54 ^a^	0.58 ^a^
Pain	0.55 ^a^	0.47 ^a^	0.49 ^a^	0.58 ^a^
Emotions	0.51 ^a^	0.45 ^a^	0.55 ^a^	0.56 ^a^
Social behavior	0.57 ^a^	0.47 ^a^	0.67 ^a^	0.64 ^a^
SF-36				
Physical functioning	0.56 ^a^	0.56 ^a^	0.67 ^a^	0.67 ^a^
Physical role limitations	0.43 ^a^	0.34 ^b^	0.35 ^ab^	0.43 ^a^
Bodily pain	0.44 ^a^	0.39 ^b^	0.49 ^a^	0.49 ^a^
General health perception	0.31 ^b^	0.38 ^b^	0.33 ^b^	0.37 ^b^
Vitality	0.41 ^a^	0.47 ^a^	0.50 ^a^	0.50 ^a^
Social functioning	0.46 ^a^	0.48 ^a^	0.45 ^a^	0.51 ^a^
Emotional role limitations	0.48 ^a^	0.48 ^a^	0.41 ^a^	0.51 ^a^
Mental health	0.33 ^b^	0.36 ^b^	0.37 ^b^	0.39 ^a^
Total score	0.55 ^a^	0.56 ^a^	0.60 ^a^	0.64 ^a^

**Table 6:** Tertiles of the total WIQ score compared with the mean maximum walking distance (MWT) and the mean initial claudication distance (InCD).

WIQ Total Score (Terciles)	n	MWD [m] (SD)	InCD [m] (SD)
0–16.33	40	195.55 (91.25)	58.15 (42.63)
16.34–18.66	32	265.59 (80.66)	83.10 (61.07)
18.66–22	31	309.10 (97.52)	139.45 (97.44)

## Data Availability

The original contributions presented in this study are included in the article/Supplementary Material. Further inquiries can be directed to the corresponding author.

## Supplementary Materials

The following supporting information can be downloaded at: https://www.mdpi.com/article/10.3390/medsci14040437/s1, File S1: Polish version of WIQ.

## Abbreviations

The following abbreviations are used in this manuscript:

6 MWT	six-minute walk test
ABI	ankle–brachial index
ACD	absolute claudication distance
HRQoL	health-related quality of life
IC	intermittent claudication
InCD	initial claudication distance
MWD	maximum walking distance
MWT	maximum walking time
PAD	peripheral arterial disease
SF-36	The 36-Item Medical Outcomes Study Short Form
TBI	toe–brachial index
QoL	quality of life
WIQ	Walking Impairment Questionnaire
