# Supplementary material for: Linguistic and Clinical Validation of the Polish Version of the Walking Impairment Questionnaire
Source: Med Sci (Basel). 2026 Jul 26;14(4):437. doi: 10.3390/medsci14040437 (PMC13515876; doi:10.3390/medsci14040437)
Supplement: Supplementary file 1 [file medsci-14-00437-s001.zip › medsci-4362639-supplementary.pdf]

Numer protokołu	Numer uczestnika	Data wizyty (dd-mm-rr)
		Wizyta ____

Czas: (zegar 24-godzinny)

Kwestionariusz dotyczący problemów z chodzeniem

Kwestionariusz do ustnego zastosowania przez personel badawczy.

Poniższe pytania dotyczą powodów dlaczego miał/a Pan/Pani trudności z chodzeniem. Chcielibyśmy dowiedzieć się, jak dużo trudności, w ciągu ostatniego miesiąca, miał/a Pan/Pani z chodzeniem z powodu każdego z niżej wymienionych problemów. Poprzez trudność należy rozumieć to, z jakim trudem bądź wysiłkiem fizycznym wiązało się dla Pana/Pani chodzenie z powodu każdego z tych problemów. Za pomocą znaku „X" proszę zaznaczyć kwadrat wskazujący najlepszą odpowiedź.

A. PYTANIA DOTYCZĄCE CHOROBY TĘTNIC OBWODOWYCH	Noga	STOPIEŃ TRUDNOŚCI				
Za pomocą znaku „X" proszę zaznaczyć kwadrat jeśli ból dotyczy lewej nogi, prawej nogi lub obu.	☐ Prawa<br>☐ Lewa<br>☐ Obie					
		ŻADEN	MAŁY	TROCHĘ	DUŻY	OLBRZYMI
1. Ból, pobolewanie lub skurcze w łydkach (lub pośladkach)?		4	3	2	1	0

B. ROZPOZNANIE RÓŻNICOWE	STOPIEŃ TRUDNOŚCI				
	ŻADEN	MAŁY	TROCHĘ	DUŻY	OLBRZYMI
1. Ból, sztywność lub pobolewanie w stawach (skokowych, kolanowych lub biodrowych)?	4	3	2	1	0
2. Osłabienie w jednej lub obu nogach?	4	3	2	1	0
3. Ból lub dyskomfort w klatce piersiowej?	4	3	2	1	0
4. Zadyszka?	4	3	2	1	0
5. Palpitacja serca?	4	3	2	1	0
6. Inne problemy	4	3	2	1	0

Numer protokołu	Numer uczestnika	Data wizyty (dd-mm-rr)
		Wizyta ____

Kwestionariusz dotyczący problemów z chodzeniem

2. ODLEGŁOŚĆ DO PRZEJŚCIA: Proszę określić stopień trudności fizycznej, który najlepiej opisuje to, jak ciężko było Panu/Pani pokonać <u>każdą</u> z danych odległości chodząc po równym terenie, bez zatrzymywania się w celu odpoczynku.

ODLEGŁOŚĆ	STOPIEŃ TRUDNOŚCI				
	ŻADEN	MAŁY	TROCHĘ	DUŻY	NIEZDOLNY/A
1. Chodzenie w pomieszczeniach np. po domu?	4	3	2	1	0
2. Przejście 20 metrów?	4	3	2	1	0
3. Przejście 50 metrów?	4	3	2	1	0
4. Przejście 100 metrów?	4	3	2	1	0
5. Przejście 200 metrów?	4	3	2	1	0
6. Przejście 300 metrów?	4	3	2	1	0
7. Przejście 500 metrów?	4	3	2	1	0

3. PRĘDKOŚĆ CHODZENIA: Poniższe pytania dotyczą tego z <u>jaką prędkością</u> w ciągu ostatniego miesiąca był/a Pan/Pani w stanie przejść odległość 100 metrów po płaskiej powierzchni. Proszę określić stopień trudności fizycznej wymaganej do pokonania przez Pana/Panią tej odległości <u>przy każdej z wymienionych prędkości, bez zatrzymywania się w celu odpoczynku</u>.

PRĘDKOŚĆ	STOPIEŃ TRUDNOŚCI				
	ŻADEN	MAŁY	TROCHĘ	DUŻY	NIEZDOLNY/A
1. Przejście 100 metrów powoli?	4	3	2	1	0
2. Przejście 100 metrów w Pana/i średnim tempie?	4	3	2	1	0
3. Przejście 100 metrów szybko?	4	3	2	1	0
4. Bieg lub trucht przez 100 metrów?	4	3	2	1	0

Numer protokołu	Numer uczestnika	Data wizyty (dd-mm-rr)
		Wizyta ____

Kwestionariusz dotyczący problemów z chodzeniem

4. WCHODZENIE PO SCHODACH: Poniższe pytania dotyczą tego ile pięter jest Pan/Pani w stanie pokonać. Proszę określić stopień trudności fizycznej jakiej doświadczył/a Pan/Pani podczas wchodzenia po schodach, na każdą wysokość podaną w pytaniach, bez zatrzymywania się w celu odpoczynku.

PIĘTRA	STOPIEŃ TRUDNOŚCI				
	ŻADEN	MAŁY	TROCHĘ	DUŻY	NIEZDOLNY/A
1. Wchodzenie na 1 piętro?	4	3	2	1	0
2. Wchodzenie na 2 piętro?	4	3	2	1	0
3. Wchodzenie na 3 piętro?	4	3	2	1	0

Nazwisko osoby przeprowadzającej ankietę: ______________________________

Podpis osoby przeprowadzającej ankietę: ______________________________

Data: ___ ___ / ___ ___ / ___ ___
(dd-mm-rr)
